# Intra-Individual Variability of Myocardial Blood Flow and Flow Reserve Assessed by [^15^O]H_2_O-PET in Patients with Angina and No Obstructive Coronary Disease

**DOI:** 10.3390/diagnostics16131975

**Published:** 2026-06-25

**Authors:** Laila Seidelin, Eva Prescott, Mads Fischer, Rasmus Haahr, Peter Hovind, Maira Rauf, Martin Krakauer

**Affiliations:** 1 Department of Cardiology, Copenhagen University Hospital, Bispebjerg and Frederiksberg Hospital, Bispebjerg Bakke 23, 2400 Copenhagen, Denmark; eva.irene.bossano.prescott@regionh.dk (E.P.); mf@nexs.ku.dk (M.F.); rasmus.haahr@regionh.dk (R.H.); maira.rauf@regionh.dk (M.R.); 2 Department of Clinical Medicine, University of Copenhagen, 2200 Copenhagen, Denmark; 3 Department for Nutrition, Exercise and Sports, University of Copenhagen, 2100 Copenhagen, Denmark; 4 Department of Clinical Physiology and Nuclear Medicine, Copenhagen University Hospital, Bispebjerg and Frederiksberg Hospital, Bispebjerg Bakke 23, 2400 Copenhagen, Denmark; peter.hovind@regionh.dk (P.H.); martin.krakauer@regionh.dk (M.K.)

**Keywords:** [^15^O]H_2_O, positron emission tomography, myocardial blood flow, reproducibility, test–retest variability, myocardial flow reserve

## Abstract

**Background/Objectives:** Myocardial blood flow (MBF) and myocardial flow reserve (MFR) are key measurements in myocardial perfusion imaging (MPI), with [^15^O]H_2_O-PET considered the reference standard. To further establish clinical and research utility, we investigated intra-individual variability of MBF and MFR over time in patients with angina, but no obstructive coronary disease. **Methods:** In a routine clinical setting, we prospectively studied 21 patients, >50 years with normal left ventricular function and no known coronary stenosis, undergoing clinically indicated PET MPI. Scan and re-scan were conducted within 30 days. Analyses were conducted by general clinical staff and re-evaluated by an expert reader. Reproducibility was assessed using paired *t*-tests, Bland–Altman analysis, repeatability coefficient (RC), within-subject coefficient of variation (CV) and intraclass correlation coefficient (ICC). **Results:** Twenty-one patients (mean age 70 ± 8.2 years; 48% female) underwent repeated scans with a median interval of 21 days. Resting MBF was 0.91 ± 0.24 vs. 0.92 ± 0.22 mL/min/g (r = 0.87; RC 0.23 mL/min/g; CV 9%; ICC 0.87). Hyperaemic MBF averaged 3.06 ± 0.9 vs. 2.97 ± 0.78 mL/min/g (r = 0.83; RC 0.98 mL/min/g; CV 11.6%; ICC 0.81). MFR showed only moderate reproducibility (3.47 ± 1.23 vs. 3.23 ± 0.92; RC 1.90; CV 21%; ICC 0.60). Neither expert re-evaluation nor rate–pressure product correction of the resting MBF improved the reproducibility. Variability was largely unaffected by atrial fibrillation and diurnal variation. **Conclusions:** Resting and hyperaemic MBF are reproducible, whereas MFR shows moderate variability, which should be considered when interpreting changes in individual patients.

## 1. Introduction

Myocardial flow reserve (MFR) is an independent biomarker of coronary vasodilatory capacity integrating macro- and microvascular circulation. It is the ratio of peak to resting myocardial blood flow (MBF), reflecting the integrated vasodilatory capacity of the epicardial and microvascular circulation [1].

In patients without obstructive coronary artery disease, reduced MFR is diagnostic of coronary microvascular dysfunction (CMD) and independently associated with adverse cardiovascular outcomes [2,3,4,5].

Positron emission tomography (PET)-based myocardial perfusion imaging (MPI) is the reference standard for non-invasive quantification of myocardial blood flow and MFR [6].

Among available tracers, [^15^O]H_2_O is regarded as the reference standard due to its near-complete extraction and linear relationship between tracer uptake and myocardial blood flow [7,8,9], allowing accurate assessment across a wide range of flow values. Consequently, [^15^O]H_2_O-PET is increasingly used in clinical practice for guiding revascularization decisions, and for evaluation of coronary microvascular function in patients without obstructive coronary artery disease [10].

Despite its established theoretical and technical validity, data on the reproducibility of [^15^O]H_2_O-PET-derived myocardial perfusion measurements in routine clinical populations are limited. Prior reproducibility studies have largely been conducted under highly standardized conditions in small cohorts of healthy volunteers, limiting their generalizability to everyday clinical practice [11,12].

The aim of this study was to evaluate the intra-individual reproducibility of resting and stress-induced myocardial perfusion measurements obtained with [^15^O]H_2_O-PET in a routine clinical setting. We hypothesized the absence of systematic bias between repeated scans and assessed reproducibility using the repeatability coefficient and the within-subject coefficient of variation.

## 2. Methods

### 2.1. Study Population

Patients who had undergone clinically indicated [^15^O]H_2_O MPI at Copenhagen University Hospital, Bispebjerg from April 2024 to February 2025 due to angina or angina equivalents were invited to undergo a repeat scan.

### 2.2. Eligibility Criteria

Participants were eligible if they were over 50 years of age and had a normal left ventricular ejection fraction (>45%) documented by an echocardiogram within the previous year.

Exclusion criteria included known ischemic heart disease or significant regional perfusion defects on imaging, as—in the absence of obstructive CAD—the PET measurements are considered a direct indicator of the microcirculation [13,14,15] (according to routine reading of the scan using a cut-off of two adjacent myocardial segments with a hyperaemic MBF value of ≤2.3 mL/min/g) [16]. Further exclusion criteria were cardiovascular events occurring between scans, severe chronic obstructive pulmonary disease or asthma, claustrophobia, acute severe illness, or substantial language barriers.

All participants gave written consent for participation and publication after receiving oral and written information according to the Helsinki declaration. The study was approved by the Regional Committee of Health Research Ethics for the Capital Region of Denmark (H-23070312).

### 2.3. PET-CT Scans and Imaging Protocol

Scans were performed on separate days with the interval kept as short as possible, not exceeding 30 days, to minimize potential changes in lifestyle and/or medication. Patients were instructed to abstain from caffeine and theophylline-containing food, beverages or medication for 24 h prior examination. Phosphodiesterase type 5 inhibitors were discontinued for five days prior to examination, drugs containing dipyridamole or nicorandil for two days, extended-release nitrates for 12 h, and short-acting nitrates for 2 h.

All examinations were conducted using a standard rest–stress protocol. Prior to the examination, participants rested on the scanner bed for 5 min, after which, systolic and diastolic blood pressure and heart rate were recorded. The examination was initiated with a CT scan for attenuation correction. A 5 min dynamic PET acquisition in list mode was performed under resting conditions, initiated simultaneously with an intravenous bolus injection of approximately 600 MBq [^15^O]H_2_O, supplied via a bedside automated system (Hidex RWG, Hidex Oy, Turku, Finland). Subsequently, myocardial perfusion was assessed under pharmacologically induced vasodilation by intravenous adenosine infusion (140 μg/kg/min for 6 min). Adenosine infusion was initiated 2 min before stress imaging to ensure the presence of maximal hyperaemia.

Although the same acquisition protocol was used, operators and day-to-day setup were not controlled; therefore, the reported variability reflects within-site reproducibility under real-world conditions rather than strict technical repeatability.

All examinations were carried out on a Discovery 710 PET-CT scanner (GE Healthcare, Milwaukee, WI, USA).

### 2.4. Diurnal Variation and Atrial Fibrillation

To assess potential variation related to the time of day, scans were categorized as AM or PM. We evaluated whether myocardial flow variation reflected diurnal effects by comparing similar (morning–morning, afternoon–afternoon) versus different times of day. In patients with atrial fibrillation, we compared patients with atrial fibrillation to patients without. All patients with atrial fibrillation had atrial fibrillation on both scan days.

### 2.5. Analysis and Readers

[^15^O]H_2_O MPI analyses of both global resting and hyperaemic MBF were performed using the Carimas CE (CarimasCE software (version 1.3.8; Varsinais-Suomen hyvinvointialue, Turku, Finland)), which has previously demonstrated excellent technical reproducibility and agreement [17].

All examinations, including image acquisition and initial readings, were conducted by routine clinical staff. All scans were subsequently evaluated by an experienced reader (expert) with more than 15 years of experience in nuclear cardiology. The expert reader was blinded to clinical information and the routine clinical staff analysis and was provided with anonymized datasets in random order. To exclude epicardial obstructive disease, clinical data and regional perfusion defects on [^15^O]H_2_O MPI were assessed. Resting MBF and MFR results are presented both as unadjusted values and corrected for resting rate–pressure product (RPP), normalizing to RPP 10,000 [18].

### 2.6. Statistics

Data are reported as count (*n*) and percent (%), mean and standard deviation (SD), or median and interquartile range (IQR), as appropriate. Repeated scans were compared using a paired *t*-test. For baseline and stress flow values and for the calculated MFR, linear association between scans was assessed using Pearson’s correlation coefficient and linear regression. As these methods assess association rather than agreement, agreement between scans was evaluated using Bland–Altman analysis. Scatter plots with linear regression and Bland–Altman plots were generated.

Reproducibility was primarily assessed by calculating the repeatability coefficient (RC) and the within-subject coefficient of variation (CV), which quantify measurement variability and repeatability between scans. RC was calculated as follows: RC = 1.96 × SD_scan difference_ [19].

The within-subject CV was calculated as follows: CV = (SD_with-in subject_/mean) × 100. Within-subject CV was calculated using the square root of half the variance of paired differences divided by the overall mean of paired measurements. Comparisons were made using *t*-tests, and the Wilcoxon rank sum test was used if differences were not normally distributed.

As a secondary metric of agreement, the intraclass correlation coefficient (ICC) was assessed using ICC(2,1), calculated with the *psych* package in R (version 4.5.0). ICC values were interpreted as poor (<0.40), fair (0.40–0.59), good (0.60–0.74), or excellent (≥0.75) agreement [20,21].

The sample size was chosen to estimate RC and CV with reasonable precision, rather than to detect small systematic differences, and was based on previous PET-CT repeatability studies [12,22,23,24], which included 15–20 participants, as well as practical considerations.

## 3. Results and Analysis

### 3.1. Study Population

Twenty-one patients were included. The baseline characteristics of the study group are given in Table 1. The median interval between scans was 21 days [range 10–29]. Heart rate and blood pressure at rest and stress conditions were similar, and were comparable between sexes across both scans, with no significant differences (Supplementary Table S1).

Due to technical problems, one patient had the first resting measurement excluded, and due to movement artefacts, another participant’s second hyperaemic MBF measurement was unusable and had to be excluded. Therefore, there are 20 comparable measurements for both resting and hyperaemic measurements: Hence, there are 19 comparable MFR values.

### 3.2. Resting MBF

**Figure 1:**
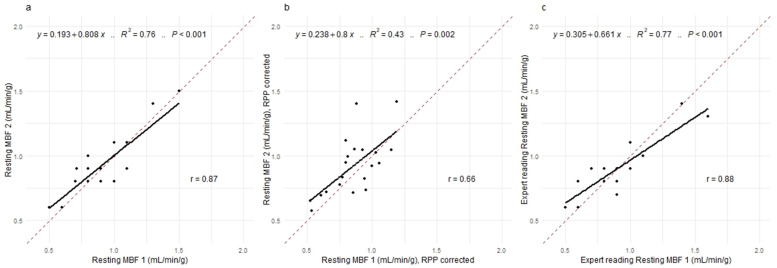
(**a**–**c**) resting myocardial blood flow reproducibility by scatter plots. (**a**) Scan–rescan comparison of the original resting myocardial blood flow measurements. (**b**) Scan–rescan comparison of the original rate–pressure product-corrected resting myocardial blood flow measurements. (**c**) Scan–rescan comparison of the expert reading of the resting myocardial blood flow measurements. Black dots represent individual measurements. The solid black line represents the linear regression line. The red dashed line represents the line of identity (y = x).

Mean resting MBF was 0.91 mL/min/g at baseline and 0.92 mL/min/g at repetition (Table 2). Resting MBF showed strong correlations between scans (r = 0.87, *p* < 0.001 for both the original and expert analyses), whereas correlations were weaker after RPP correction of the original clinical reading (r = 0.66, *p* < 0.002) (Figure 1a–c).

The repeatability coefficient (RC) was 0.23 mL/min/g for the original analysis, 0.25 mL/min/g for expert, and 0.34 mL/min/g after RPP correction.

The within-subject coefficients of variation (CVs) were 9.2% for the original clinical readings and 9.9% for the expert re-evaluations, whereas the RPP-corrected original values had a CV of 14% (Table 2).

**Figure 2:**
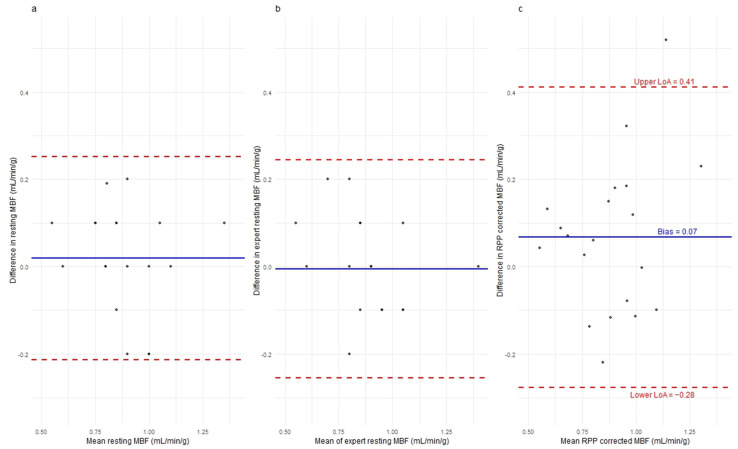
(**a**–**c**) resting myocardial blood flow reproducibility by Bland–Altman plot. (**a**) Bland–Altman analysis of resting myocardial blood flow. (**b**) Bland–Altman analysis of expert-reassessed resting myocardial blood flow. (**c**) Bland–Altman analysis of rate–pressure product-corrected resting myocardial blood flow. Black dots represent individual measurements.

Bland–Altman analysis of resting MBF showed minimal systematic bias between repeated scans (Figure 2a–c). For the original rest measurement (Figure 2a), the mean bias was 0.02 mL/min/g (95% CI −0.04 to 0.08) with limits of agreement (LoA) from −0.21 to 0.25. Expert re-evaluation yielded a similar small bias of −0.01 mL/min/g (95% CI −0.06 to 0.05) and comparable limits of agreement (−0.26 to 0.25) (Figure 2b). In contrast, RPP-adjusted resting MBF showed wider limits of agreement (−0.28 to 0.41) despite minimal bias 0.07 mL/min/g (95% CI −0.01 to 0.15) (Figure 2c). For the resting MBF, the ICC was 0,87 for the original MBF, 0.85 for the expert-evaluated MBF and 0.62 for the RPP-corrected MBF (Table 2).

Overall, original and expert-evaluated MBF demonstrated good reproducibility, whereas RPP adjustment led to greater variability.

### 3.3. Hyperaemic MBF

**Figure 3:**
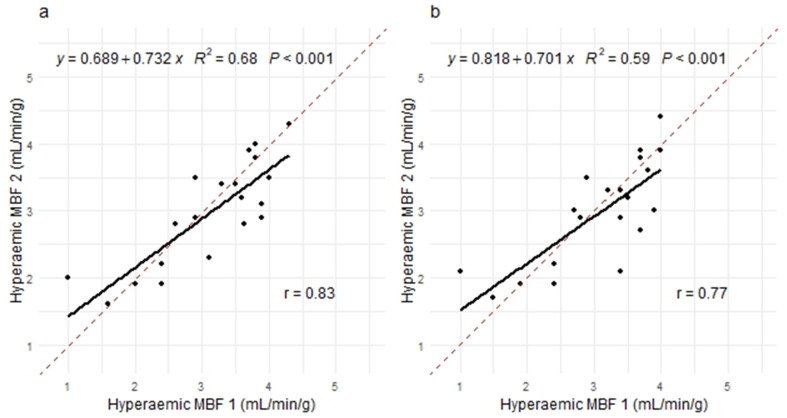
(**a**,**b**) hyperaemic myocardial blood flow reproducibility by scatter plot. (**a**) scan–rescan comparison of the original hyperaemic myocardial blood flow measurements. (**b**) scan–rescan comparison of the expert reading of hyperaemic myocardial blood flow measurements. Black dots represent individual measurements. The solid black line represents the linear regression line. The red dashed line represents the line of identity (y = x).

Mean hyperaemic MBF was 3.06 mL/min/g at baseline and 2.97 mL/min/g at repeat examination (Table 2). Hyperaemic MBF measurements were strongly correlated between scans (r = 0.83 and r = 0.77, respectively; both *p* < 0.001) (Figure 3a,b). The within-subject coefficients of variation were 11.6% for the original clinical readings and 13.1% for the expert re-evaluations, indicating good reproducibility, and the repeatability coefficient during stress was 0.98 mL/min/g for the original analysis and 1.09 mL/min/g for the expert re-evaluation (Table 2).

**Figure 4:**
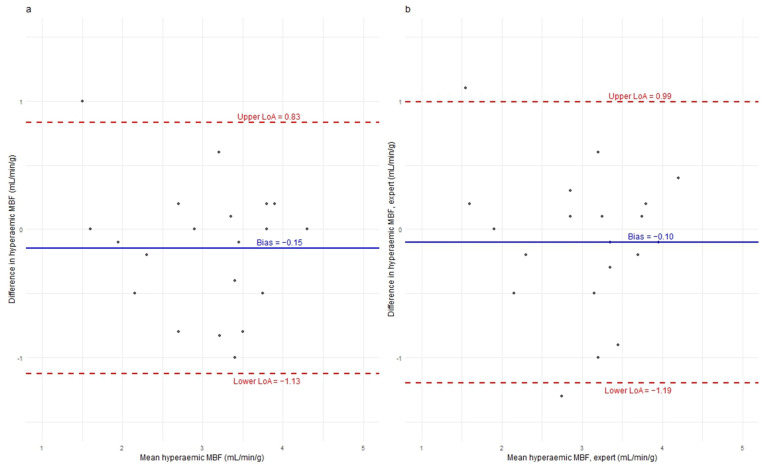
(**a**,**b**) hyperaemic myocardial blood flow reproducibility by Bland–Altman Plot. (**a**) Bland–Altman analysis of hyperaemic myocardial blood flow. (**b**) Bland–Altman analysis of the expert hyperaemic myocardial blood flow reading. Black dots represent individual measurements.

Bland–Altman analyses demonstrated minimal systematic bias between repeated stress scans, as illustrated in Figure 4a,b. For the original analysis, the mean bias was −0.15 mL/min/g (95% CI −0.38 to 0.09) with limits of agreement from −1.13 to 0.83. Expert re-evaluation showed a similarly small bias of −0.10 mL/min/g (95% CI −0.36 to 0.16) with limits of agreement from −1.19 to 0.99. The ICC was 0.81 for the original hyperaemic MBF analysis and 0.77 for the expert-re-evaluated (Table 2).

Overall, hyperaemic MBF demonstrated good reproducibility, with no evidence of systematic bias on Bland–Altman plots (Figure 4a,b).

### 3.4. Myocardial Flow Reserve

**Figure 5:**
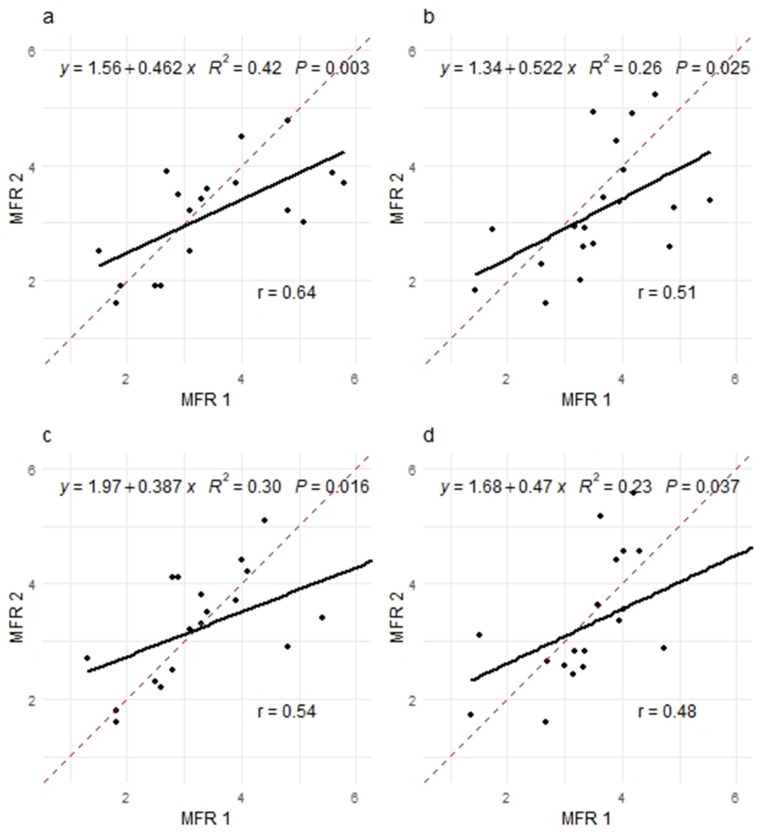
(**a**–**d**) myocardial flow reserve reproducibility by scatter plot. (**a**) Original. scan–rescan comparison of the myocardial flow reserve, calculated by the original resting and hyperaemic values. (**b**) RPP corrected. scan–rescan comparison of the myocardial flow reserve, calculated by the original rate–pressure product-corrected resting values and original hyperaemic values. (**c**) Expert assessment. scan–rescan comparison of the myocardial flow reserve, of the expert evaluated resting values and expert evaluated hyperaemic values. (**d**) Expert with RPP correction. scan–rescan comparison of the myocardial flow reserve, of the expert evaluated rate–pressure product-corrected resting values and expert evaluated hyperaemic values.

**Figure 6:**
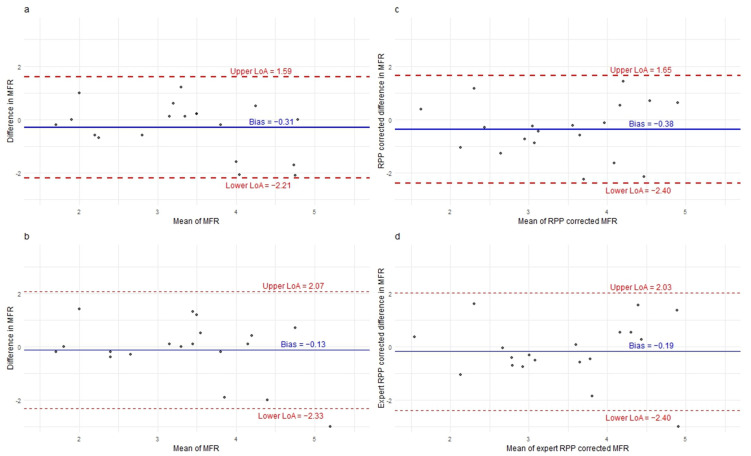
(**a**–**d**) myocardial flow reserve reproducibility by Bland–Altman plot. (**a**) Bland–Altman analysis of scan–rescan myocardial flow reserve (MFR) from original myocardial blood flow (MBF). (**b**) Bland–Altman analysis of scan–rescan MFR from expert reassessed MBF. (**c**) Bland–Altman analysis of scan–rescan MFR from rate–pressure product-corrected MBF. (**d**) Bland–Altman analysis of scan–rescan MFR from rate–pressure product-corrected expert reassessed MBF. Black dots represent individual measurements.

Mean MFR was 3.47 at baseline and 3.23 at repeat examination (Table 2). Correlations between repeated measurements were moderate across analyses (r = 0.64 for original, r = 0.51 for RPP-corrected, r = 0.54 for expert-corrected, and r = 0.48 for expert RPP-corrected; all *p* < 0.05) (Figure 5 a–d). The within-subject coefficient of variation was 21%, indicating moderate reproducibility (Table 2). Repeatability coefficients ranged from 1.90 to 2.21 across analyses, and ICC ranged from 0.60 to 0.49, with wide limits of agreement (Table 2). Bland–Altman analyses demonstrated small mean differences between repeated measurements with no evidence of systematic bias. For uncorrected MFR, the mean bias was −0.31 (CI: −0.78–0.15), with limits of agreement from −2.21 to 1.59 with similar findings for RPP-corrected and expert-derived measurements (Figure 6a–d).

Overall, MFR showed moderate reproducibility with no systematic bias on Bland–Altman plots.

### 3.5. Atrial Fibrillation and Diurnal Variation

In participants with atrial fibrillation (*n* = 5), Bland–Altman plots showed random scatter across MBF values, with no systematic bias or trend toward wider limits of agreement (Supplementary Figure S3).

There were no systematic diurnal differences in resting, hyperaemic or MFR measurements (Supplementary Figure S2).

## 4. Discussion

This is, to our knowledge, the first study to assess the test–retest reproducibility of clinically indicated [^15^O]H_2_O PET myocardial blood flow measurements. By restricting the analysis to patients without significant regional perfusion defects indicating coronary artery stenosis, we specifically assessed the reliability of coronary microvascular measurements, independent of regional ischemia. Overall, we observed the reproducibility of resting and hyperaemic myocardial blood flow, but larger variability in myocardial flow reserve. No effect of atrial fibrillation or diurnal variation was observed; however, the study was not powered to detect such effects, particularly for atrial fibrillation.

### 4.1. Resting and Hyperaemic MBF Reproducibility

In our clinical cohort, resting MBF and hyperaemic MBF demonstrated good reproducibility, with within-subject coefficients of variation of 9% and 12% and repeatability coefficients of 0.23 and 0.98 mL/min/g, respectively. Despite being conducted under routine clinical conditions, these findings closely align with repeatability estimates previously reported in controlled laboratory settings. Kaufmann et al. examined [^15^O]H_2_O-PET myocardial perfusion in 21 healthy men scanned one hour apart and reported repeatability coefficients of 0.17 mL/min/g at rest and 0.94 mL/min/g during hyperaemia [12]. Similarly, Manabe et al. studied 15 healthy individuals using ^82^Rb-PET and found repeatability coefficients of 0.19 mL/min/g for resting MBF and 0.92 mL/min/g for hyperaemic MBF [22]. Repeatability of resting MBF has also been shown to be approximately 0.20 mL/min/g for both ^82^Rb and ^13^N-NH_3_ in same-day measurements [1,25], and comparable values have been reported in a chronic coronary artery disease animal model using both [^15^O]H_2_O-PET and ^13^N-NH_3_-PET [26].

For hyperaemic MBF, a prior ^82^Rb-PET dipyridamole-based study by Kitkungvan et al. reported stress MBF test–retest variability of 10% within the same session and 21% between days [27], corresponding to estimated repeatability coefficients of 0.64 and 1.27 mL/min/g, respectively. Similarly, a Swedish study abstract using same-day [^15^O]H_2_O-PET in 10 patients with angina reported a repeatability coefficient for hyperaemic MBF of 0.43 mL/min/g, corresponding to a CV of 6% [28].

Compared with other imaging modalities, reproducibility estimates are similar. A UK study of 11 healthy subjects undergoing cardiac magnetic resonance (CMR) perfusion imaging one week apart reported CVs of 16.0% at rest and 26.8% during stress [29], while a Doppler echocardiography study in 86 patients reported CVs of 15% at rest and 17% during hyperaemia [30].

Our results demonstrate that [^15^O]H_2_O-PET MBF measures maintain high reproducibility under real-world clinical conditions, across operators and over time.

### 4.2. MFR Reproducibility

Only a limited number of studies have evaluated the reproducibility of myocardial flow reserve, whether using [^15^O]H_2_O-PET or other tracers, and these have largely been conducted in small cohorts of healthy volunteers under controlled experimental conditions [12,22]. Importantly, none have assessed reproducibility in a routine clinical setting using clinically indicated PET examinations repeated on separate days.

In our study, [^15^O]H_2_O-PET MPI-derived MFR showed a within-subject coefficient of variation of 21% and a repeatability coefficient of 1.9. In comparison, Kaufmann et al. reported an MFR repeatability coefficient of 1.3 in healthy men scanned one hour apart using [^15^O]H_2_O-PET [12], and studies using ^82^Rb-PET have reported MFR repeatability coefficients of approximately 1.6 [22]. For cardiovascular magnetic resonance perfusion imaging, MFR reproducibility has been reported with a CV of approximately 24% [29]. In contrast, Doppler-based coronary flow velocity reserve (CFVR) assessed by echocardiography has demonstrated lower variability, with a CV of 11% overall and as low as 5% when repeat examinations were performed within one week [30], highlighting the impact of shorter inter-scan intervals and controlled conditions.

The moderate reproducibility observed in our study likely reflects the combined biological and technical variability inherent in inter-session measurements performed days apart in a heterogeneous patient population. Moreover, variability in MFR is expected to be greater than for absolute flow measures, as MFR represents the ratio of hyperaemic to resting MBF and therefore accumulates variability from both components. MFR thus shows substantial inter-scan variability, and changes should therefore be interpreted with caution, particularly at the individual level.

### 4.3. Rate–Pressure Product Variability

MFR and hyperaemic MBF provide insight into both epicardial and microvascular coronary function. MFR may be misleading when resting flow is abnormally low or high [31]. Therefore, for the resting MBF, we did an analysis of the RPP-corrected values. The rationale is to normalize for abnormally high or low resting MBF, by considering the actual myocardial oxygen demand. This is, however, still controversial, as the clinical implications are unknown, and a study showed no significant difference in cardiovascular risk between patients who normalized their otherwise decreased MFR after RPP correction [18]. Manabe et al. concluded that, in ^82^Rb-PET, RPP correction did not improve the repeatability of rest MBF or MFR [22]. Another study even showed that uncorrected MFR has more prognostic value than corrected MFR in ^82^Rb-PET [32].

Hemodynamic variables are known to affect resting MBF more than hyperaemic MBF, leading to greater variability in resting MBF and, consequently MFR [33,34]. For resting MBF, reproducibility was high in both the original and expert analyses but decreased after correction for RPP. Hence, the RPP-corrected values increased variability compared to uncorrected values and did not enhance consistency between measurements. This suggests that the observed variation might largely be attributable to biological fluctuations and technical measurement imprecision rather than differences in hemodynamics. This aligns with our findings of relatively stable stress blood pressure and heart rate compared with rest, and attempts to correct for this variability using RPP-adjusted resting measurements did not improve consistency.

### 4.4. Technical and Biological Variation

Variation in MBF and MFR may be greater in patients with atrial fibrillation or due to diurnal effects. Separate analyses of scans performed at the same time of day (*n* = 12) did not indicate an improvement in reproducibility. This lines up with a study of cardiovascular magnetic resonance MPI repeatability where no significant diurnal variation in perfusion was observed [29]. Similarly, we did not observe a significant effect of atrial fibrillation on variability in our cohort. However, the study was not powered to detect such effects, precluding firm conclusions.

MBF and MFR measurements are known to be influenced by technical factors, such as choice of radiotracer and stress agent, as well as biological variables including age, sex, genetics, and hormonal cycles [1,31,35]. Previous studies have shown that hyperaemic MBF is comparable across commonly used vasodilator agents, including adenosine, regadenoson, and dipyridamole [1], and that they are comparable between tracers, when correcting for the nonlinear extraction of some of the tracers [36,37].

Despite the robustness of the imaging system and software [13], variability was greater during stress measurements. This is likely attributable to biological factors, some of which may be differences in fluid and food intake, hydration, medication, neurohumoral factors, and fluctuations in heart rate and blood pressure, as well as unknown biological factors. However, previous studies have demonstrated substantial variability even when repeat stress measurements are performed within the same session or within a few hours, suggesting that these slower-varying biological factors are unlikely to account for the majority of the observed variability [1,12,22,25,27,28].

### 4.5. Clinical Implications

A reduced MFR is independently associated with increased mortality and morbidity [38,39]. The diagnostic threshold for microvascular dysfunction is an MFR < 2.5 [16] when measured by [^15^O]H_2_O-PET, corresponding to the cut-off of MFR < 2.0 measured with ^82^Rubidium-PET [36,40]. Consequently, reliable measurement of MFR is clinically important. The least significant change was 1.9, indicating that smaller changes likely reflect measurement variability rather than true physiological change. At the individual level, this represents substantial variability relative to the diagnostic threshold and may influence the interpretation of serial measurements and baseline stratification of patients with or without microvascular dysfunction. Given the modest sample size of this study and the clinical importance of MFR, larger studies are warranted to further investigate the reproducibility of MFR measurements. It would be relevant to determine whether reproducibility differs across the range of MFR values, especially in patients with reduced MFR near clinically relevant decision thresholds.

### 4.6. Limitations

Although the sample size was modest (*n* = 21), it was sufficient to demonstrate consistent findings across analyses, while subgroup analyses may be underpowered. All scans were performed at a single centre, which may affect generalizability, although the standardized protocols used support broader applicability. The readers from the routine clinical staff were not blinded to the results of the previous [^15^O]H_2_O-MPI. Although objective image-derived measures reduce the risk of bias, blinded independent interpretation would have been preferable. To address this, the paired datasets were subsequently anonymized and reviewed in a blinded fashion by an expert reader, with variability comparable to that of the routine clinical staff. For the patient cohort, biological variation between study visits cannot be excluded, despite the relative short interval between scans. Minor medication adjustments occurred between scans (Supplementary Materials); however, these adjustments were small and are unlikely to have significantly influenced MBF.

Variability in operator experience and scan conditions reflected routine clinical practice and therefore enhances the clinical the relevance of the results; however, this may limit the generalisability to other centres or software packages. Notably, expert interpretation did not improve reproducibility compared with routine clinical analysis, suggesting that the semiautomatic analysis provided by the software is robust.

## 5. Conclusions

In conclusion, our findings demonstrate that [^15^O]H_2_O-MPI yields reproducible measurements of both resting and hyperaemic MBF in routine clinical practice, particularly at the group level. In contrast, MFR shows only moderate reproducibility, reflecting the cumulative variability of both its numerator and denominator, and rate–pressure product correction further increased variability. This emphasizes the need for cautious interpretation of MFR values, especially when evaluating changes between repeated measurements in individual patients.

## Figures and Tables

**Table 1:** Baseline characteristics.

**Patient characteristics**	***N*** = 21 (10 female/11 male)
**Age (years)**	69.8 (8.21)
**Sex (Female)**	10 (47.6%)
**BMI**	28.7 (6.31)
**Smoking status:** **Current** **Former** **Never**	1 (4.8%) 12 (57.1%) 8 (38.1%)
**Hypertension**	11 (52.4%)
**Hypercholesterolemia**	10 (47.6%)
**Diabetes type II**	4 (19.0%)
**Atrial fibrillation**	5 (23.8%)
**Medicine changes ^b^**	2 (9.5%)
**Interval between scans (days)**	21 (5.37) [10;29]
**Scan time of day similarity ^a^**	12 (57.1%)

Data are means (SD) or numbers (%), and [Range]. ^a^ Morning defined as 08–12, afternoon defined as 12–17. Scan time is considered similar if both scans are performed at the same time-interval. ^b^ For details regarding medicinal changes, see Supplementary Comment S1.

**Table 2:** Scan reproducibility: resting and hyperaemic myocardial blood flow and myocardial flow reserve.

	Scan 1 Mean (SD)	Scan 2 Mean (SD)	*p*-Value ^d^	Mean Difference (SD)	RC *, mL/min/g (95% CI)	CV **, % (95% CI)	ICC *** (95% CI)	Difference Median (IQR) [Range]
**Resting MBF, Global ^a^**								
**Original**	0.91 (0.24)	0.92 (0.22)	0.47	0.02 (0.12)	0.23 (0.18;0.34)	9.2 (7.0;13.4)	0.87 (0.71;0.95)	0 (0.1) [0.4]
**Expert**	0.92 (0.26)	0.91 (0.19)	0.86	−0.01 (0.13)	0.25 (0.19;0.37)	9.9 (7.5;14.4)	0.85 (0.67;0.94)	0 (0.2) [0.5]
**RPP-corrected ^c^**	0.85 (0.19)	0.92 (0.22)	0.10	0.07 (0.18)	0.34 (0.26;0.50)	14.0 (10.6;20.4)	0.62 (0.27;0.83)	0.07 (0.24) [0.7]
**Hyperaemic MBF, Global ^a^**								
**Original**	3.06 (0.90)	2.97 (0.78)	0.21	−0.15 (0.50)	0.98 (0.75;1.43)	11.6 (8.8;17.0)	0.81 (0.60;0.92)	−0.05 (0.625) [2]
**Expert**	3.0 (0.88)	2.97 (0.78)	0.21	−0.10 (0.56)	1.09 (0.83;1.60)	13.1 (10.0;19.1)	0.77 (0.51;0.90)	−0.05 (0.55) [2.4]
**Myocardial Flow Reserve ^b^**								
**Original**	3.47 (1.23)	3.23 (0.92)	0.18	−0.31 (0.97)	1.90 (1.43;2.81)	20.6 (15.5;30.4)	0.60 (0.23;0.82)	−0.02 (0.85) [3.3]
**RPP-corrected ^c^**	3.59 (1.01)	3.26 (1.05)	0.13	−0.38 (1.03)	2.03 (1.53;3.00)	21.5 (16.2;31.8)	0.49 (0.08;0.79)	−0.31 (1.42) [3.68]
**Expert**	3.40 (1.28)	3.23 (0.92)	0.63	−0.13 (1.12)	2.20 (1.66;3.25)	23.7 (17.9;35.0)	0.53 (0.12;0.79)	0 (0.7) [4.4]
**Expert RPP-corrected ^c^**	3.52 (1.09)	3.36 (1.07)	0.48	−0.19 (1.13)	2.21 (1.67;3.27)	23.3 (17.6;34.4)	0.49 (0.07;0.77)	−0.33 (1.09) [4.63]

Data are means (SD) or numbers (%), and [Range]. ^a^ Myocardial blood flow. Resting and hyperaemic flow values are in mL/min/g. ^b^ Myocardial flow reserve = hyperaemic MBF/resting MBF. ^c^ Rate–pressure product-corrected resting MBF was calculated as (resting MBF/(resting systolic blood pressure × resting heart rate)) × 10,000. ^d^ by paired *t*-test. * Repeatability coefficient = 1.96 × SD^difference^, given in mL/min/g. ** Within-subject coefficient of variation = (SD^within/^mean) × 100, given in percent. *** Intraclass correlation coefficient (ICC(2,1)).

## Data Availability

The data presented in this study are available on request from the corresponding author due to privacy. All data were handled according to the regulations by the Danish Data Protection Agency (ID: p-2023-15054).

## Supplementary Materials

The following supporting information can be downloaded at: https://www.mdpi.com/article/10.3390/diagnostics16131975/s1, Figure S1: Flowchart of screening and inclusion; Table S1: Scan characteristics; Comment S1: Medical changes during the intervention period. Figure S2: A–D: Scan–time comparison. Figure S3: A,B: Bland–Altman plots by atrial fibrillation status.

## Abbreviations

The following abbreviations are used in this manuscript:

CFVR	Coronary Flow Velocity Reserve
CV	Coefficient of Variation
CMR	Cardiac Magnetic Resonance
LoA	Limit of Agreement
MBF	Myocardial Blood Flow
MFR	Myocardial Flow Reserve
MPI	Myocardial Perfusion Imaging
PET	Positron Emission Tomography
RC	Repeatability Coefficient
RPP	Rate–Pressure Product
