# Supplementary material for: Intra-Individual Variability of Myocardial Blood Flow and Flow Reserve Assessed by [15O]H2O-PET in Patients with Angina and No Obstructive Coronary Disease
Source: Diagnostics (Basel). 2026 Jun 25;16(13):1975. doi: 10.3390/diagnostics16131975 (PMC13360267; doi:10.3390/diagnostics16131975)

Supplementary

Figure S1: Flowchart of screening and inclusion

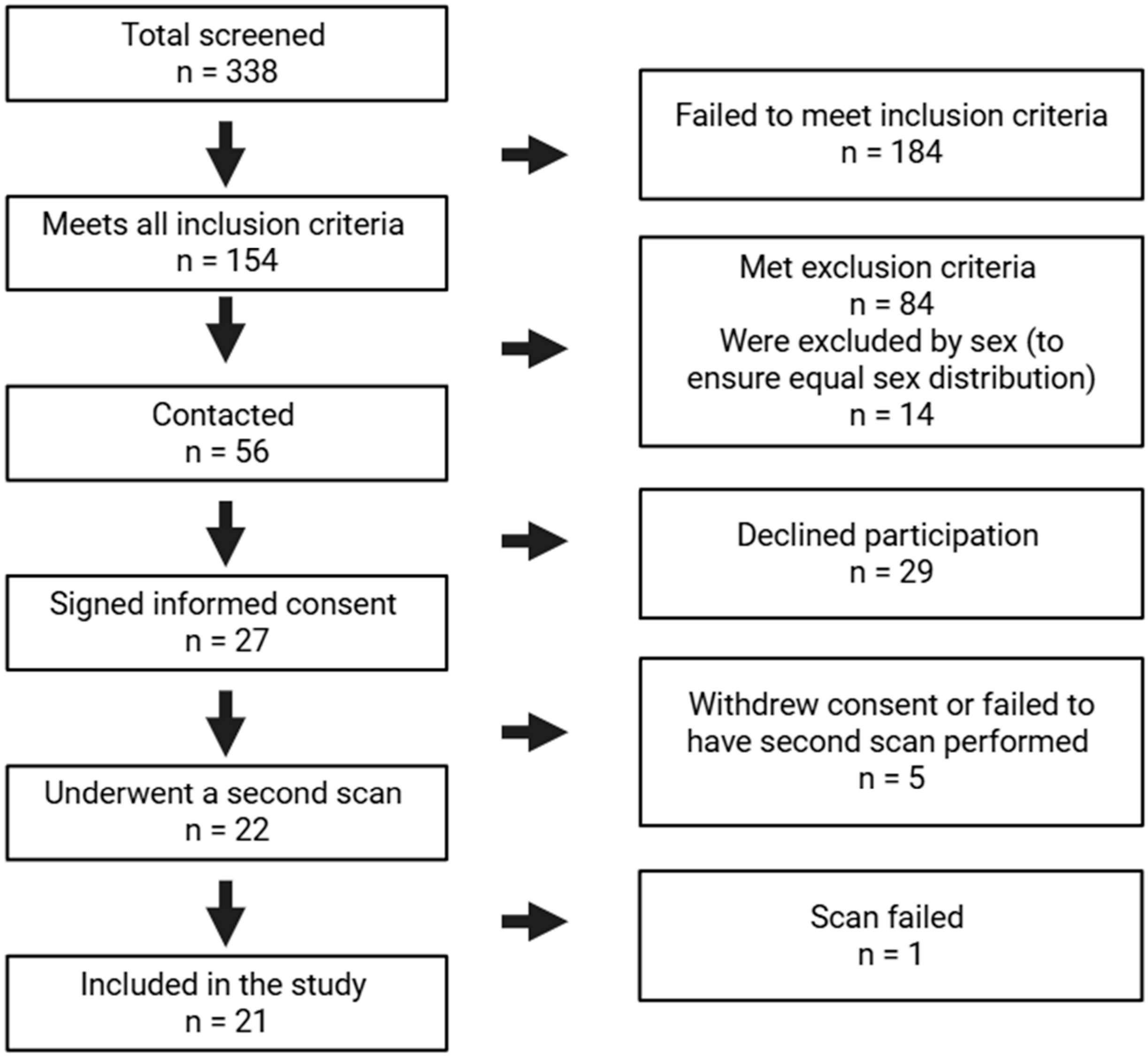

Table S1: Scan characteristics

**Scan characteristics**				
	**Scan 1**	**Scan 2**	**Δ-value**	**p-value***
Heart Rate, Rest *(beats/min)*	75.1 (12.6)	72.3 (8.7)	-2.5 (9.0) [-23;11]	0.23
BP, Systolic, Rest *(mmHg)*	143.2 (18.4)	141.0 (17.8)	-2.4 (17.9) [-34;33]	0.56
Heart Rate, Stress *(beats/min)*	75.8 (14.7)	74.5 (11.1)	-1.3 (8.5) [-16;13]	0.48
BP, Systolic, Stress *(mmHg)*	128.3 (16.0)	127.3 (14.3)	-1 (10.8) [-17;27]	0.68

Data are means (SD) or numbers (%), and [Range]

*P-values calculated by paired t-test

Δ, Delta. Mean difference between 1st and 2nd scan

Comment S1: medicinal changes during the intervention period

One participant underwent unsuccessful Direct Current Cardioversion for atrial fibrillation but remained in atrial fibrillation during both scans. Another was prescribed sublingual nitroglycerin spray but did not use it. One had Centyl (Bendroflumethiazide with potassium chloride) treatment paused, and one discontinued colchicine after a short course for suspected pericarditis and underwent an uncomplicated colonoscopy within the study timeframe.

Figure S2 A-D: Scan–time comparison:

S2A:

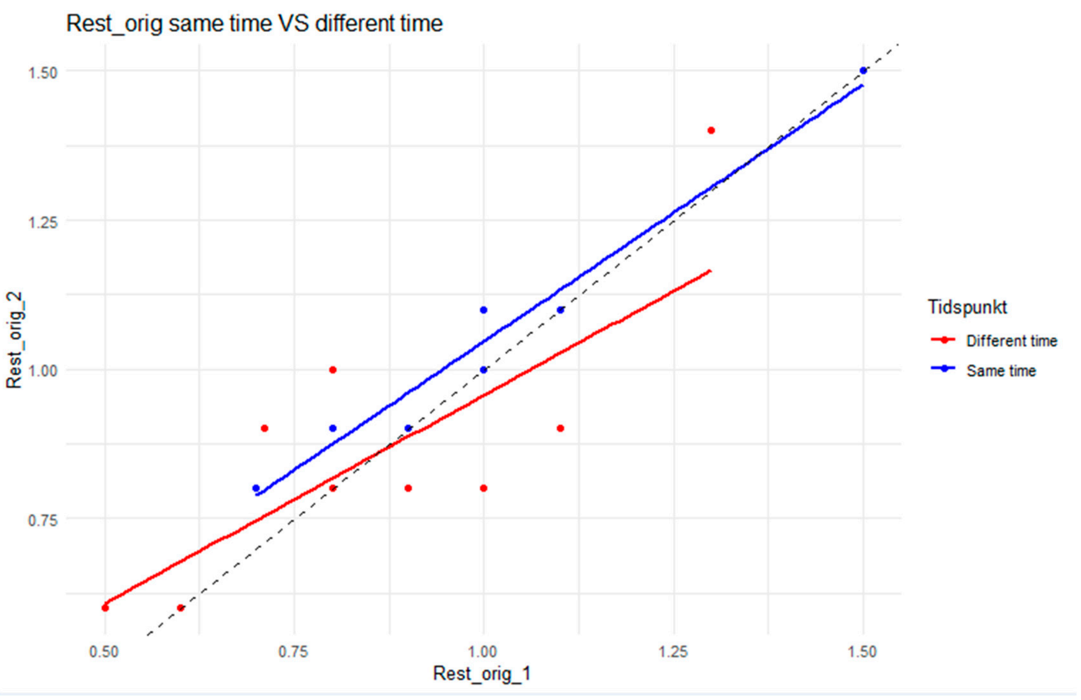

S2B:

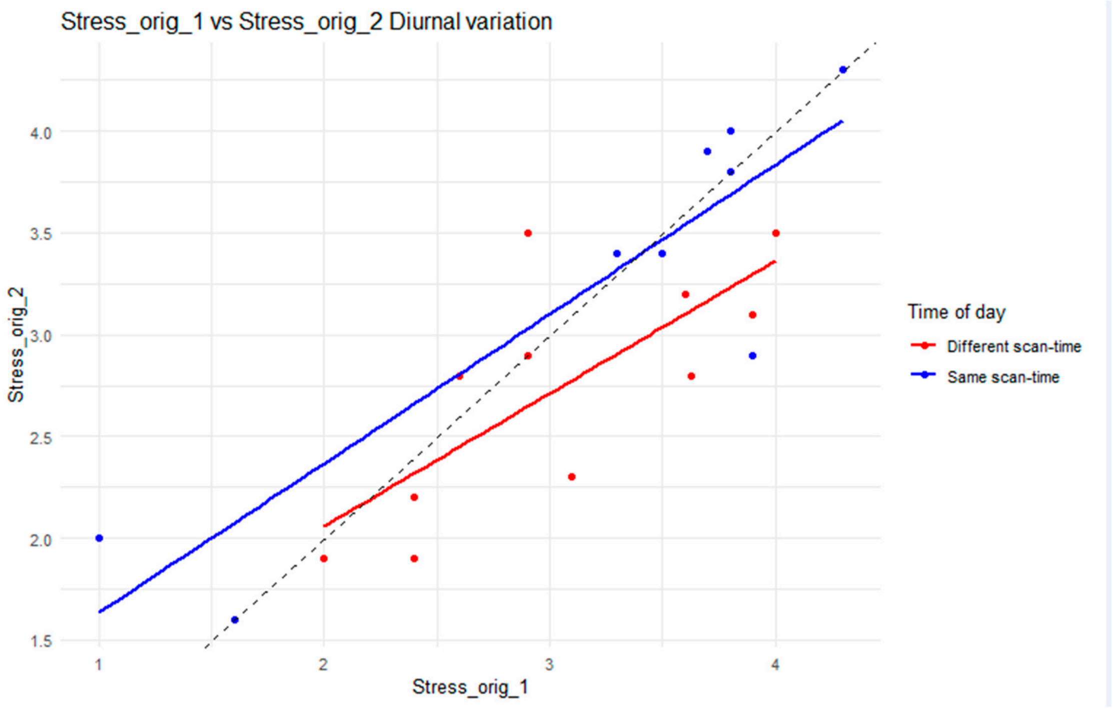

S2C:

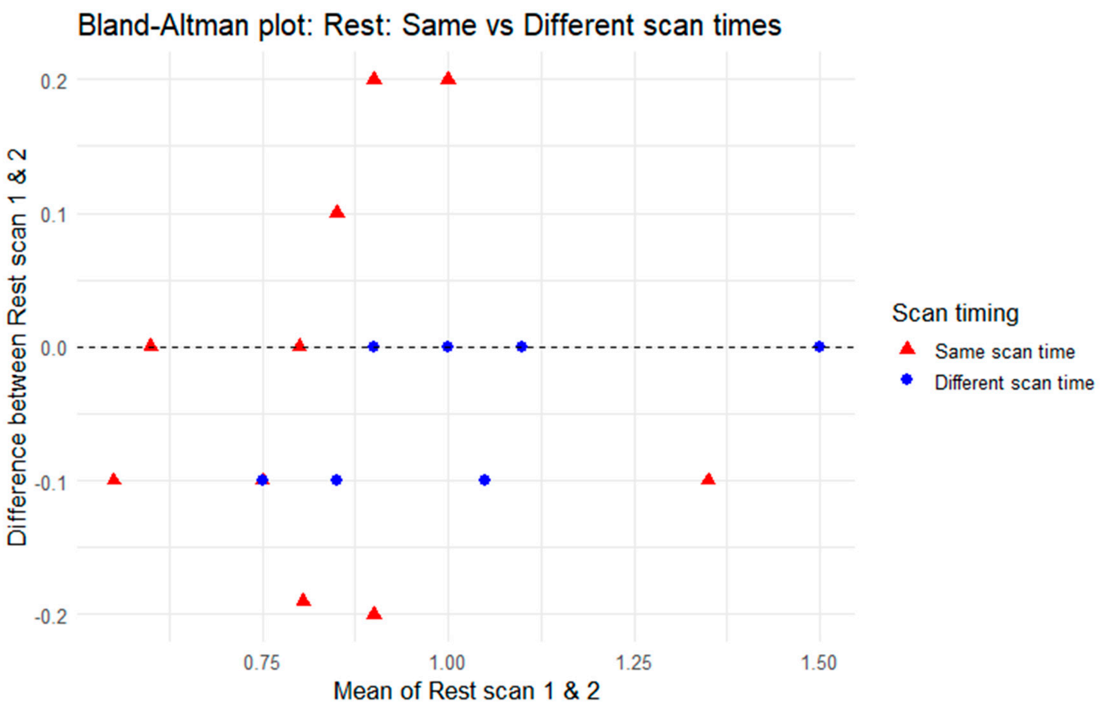
Bland-Altman plot: Rest: Same vs Different scan times
Difference between Rest scan 1 & 2
0.2
0.1
0.0
-0.1
-0.2
0.75
1.00
1.25
1.50
Mean of Rest scan 1 & 2
Scan timing
Same scan time
Different scan time

S2D:

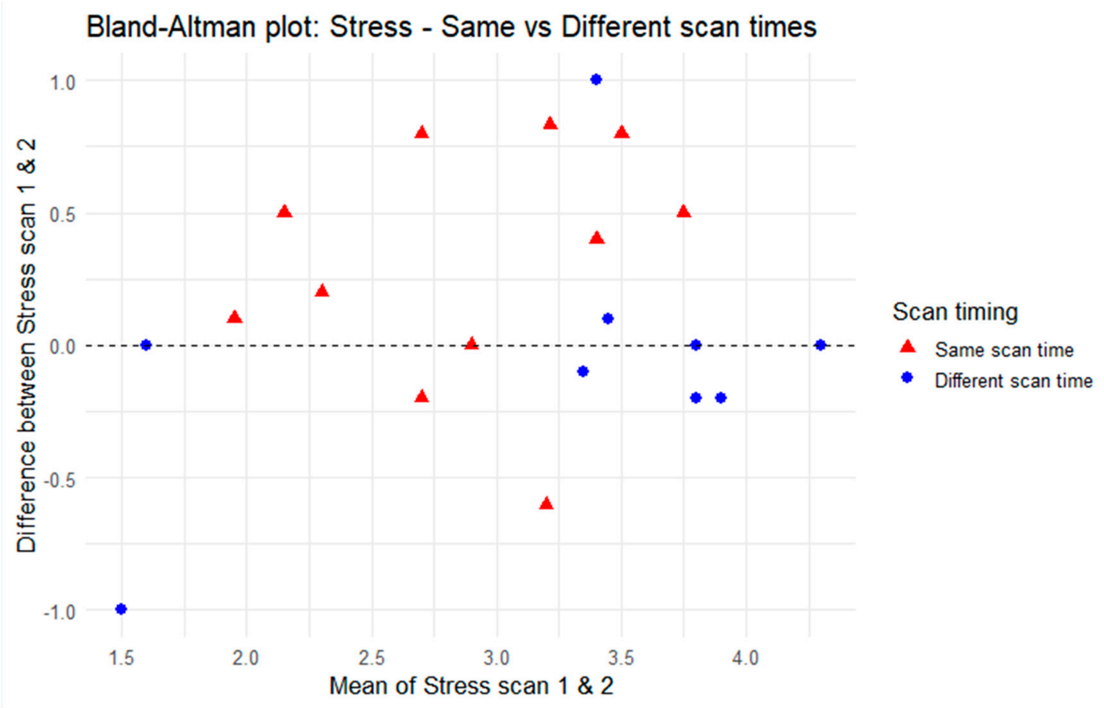
Bland-Altman plot: Stress - Same vs Different scan times
Difference between Stress scan 1 & 2
1.0
0.5
0.0
-0.5
-1.0
1.5
2.0
2.5
3.0
3.5
4.0
Mean of Stress scan 1 & 2
Scan timing
Same scan time
Different scan time

Figure S3 A and B: Bland–Altman plots by atrial fibrillation status

Figure S3A:

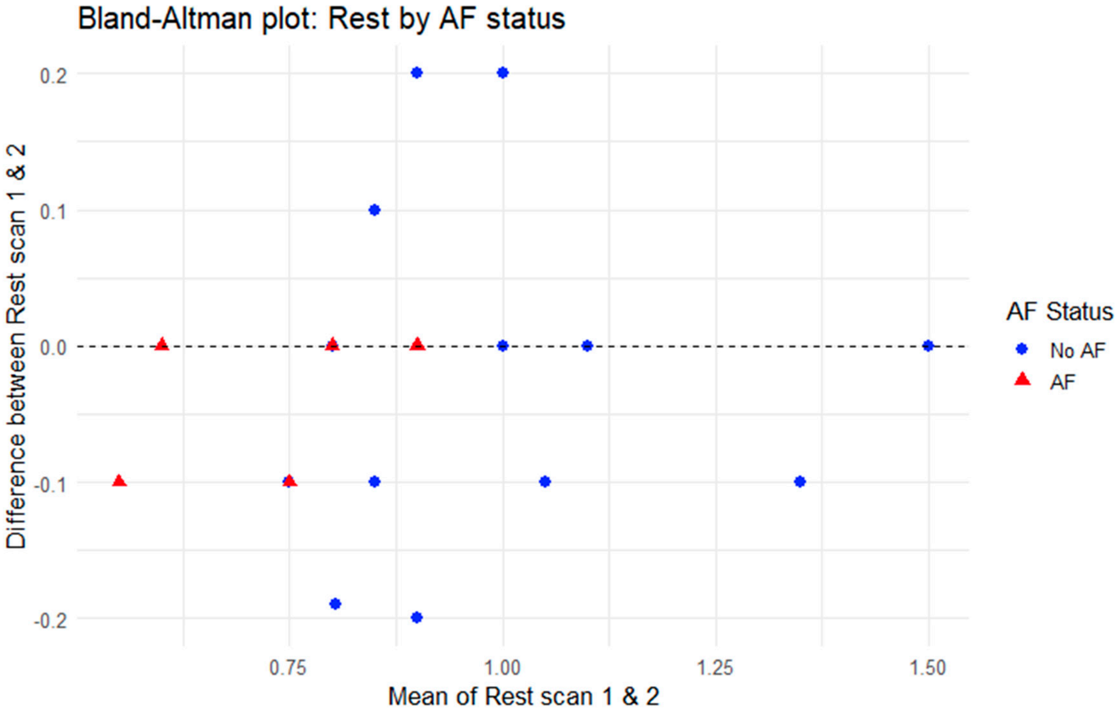

Figure S3B:

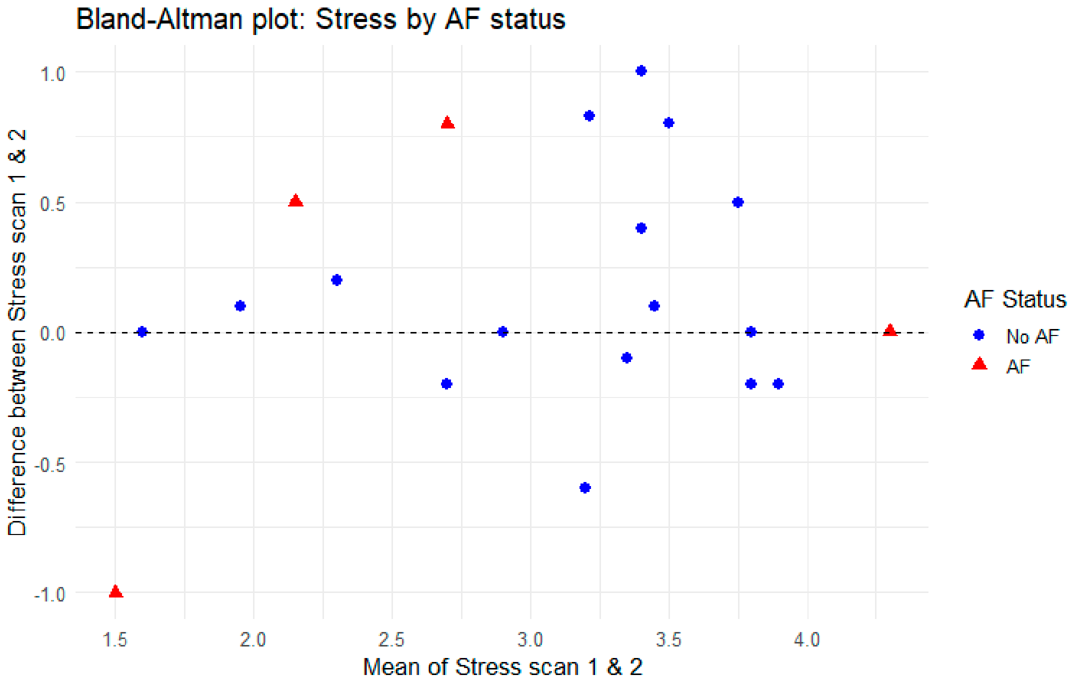

Supplement: Supplementary file 1 [file diagnostics-16-01975-s001.zip › diagnostics-4323807-supplementary.pdf]
